# Risky Behavior in Gambling Tasks in Individuals with ADHD – A Systematic Literature Review

**DOI:** 10.1371/journal.pone.0074909

**Published:** 2013-09-13

**Authors:** Yvonne Groen, Geraldina F. Gaastra, Ben Lewis-Evans, Oliver Tucha

**Affiliations:** Department of Clinical and Developmental Neuropsychology, University of Groningen, Groningen, The Netherlands; Inserm, France

## Abstract

**Objective:**

The aim of this review was to gain insight into the relationship between Attention deficit hyperactivity disorder (ADHD) and risky performance in gambling tasks and to identify any potential alternate explanatory factors.

**Methods:**

PsycINFO, PubMed, and Web of Knowledge were searched for relevant literature comparing individuals with ADHD to normal controls (NCs) in relation to their risky performance on a gambling task. In total, fourteen studies in children/adolescents and eleven studies in adults were included in the review.

**Results:**

Half of the studies looking at children/adolescents with ADHD found evidence that they run more risks on gambling tasks when compared to NCs. Only a minority of the studies on adults with ADHD reported aberrant risky behavior. The effect sizes ranged from small to large for both age groups and the outcome pattern did not differ between studies that applied an implicit or explicit gambling task. Two studies demonstrated that comorbid oppositional defiant disorder (ODD) and conduct disorder (CD) increased risky behavior in ADHD. Limited and/or inconsistent evidence was found that comorbid internalizing disorders (IDs), ADHD subtype, methylphenidate use, and different forms of reward influenced the outcomes.

**Conclusion:**

The evidence for increased risky performance of individuals with ADHD on gambling tasks is mixed, but is stronger for children/adolescents with ADHD than for adults with ADHD, which may point to developmental changes in reward and/or penalty sensitivity or a publication bias for positive findings in children/adolescents. The literature suggests that comorbid ODD/CD is a risk factor in ADHD for increased risky behavior. Comorbid IDs, ADHD subtype, methylphenidate use, and the form of reward received may affect risky performance in gambling tasks; however, these factors need further examination. Finally, the implications of the findings for ADHD models and the ecological validity of gambling tasks are discussed.

## Introduction

Attention deficit hyperactivity disorder (ADHD) is characterized by attentional problems, hyperactivity, and impulsivity [1]. Based on these symptoms, three ADHD subtypes can be distinguished: the ADHD combined type (ADHD-C), the ADHD inattentive type (ADHD-I), and the ADHD hyperactive-impulsive type (ADHD-H). The prevalence of ADHD in the general population has been estimated at 5.3% for individuals below 18 years of age and at 4.4% for adults [2], [3]. ADHD symptoms often decline during adolescence (remittent ADHD), therefore, only a portion of children with ADHD still meet all the DSM-IV criteria for ADHD when they reach adulthood (persistent ADHD) [1], [4]. Individuals with ADHD have often been found to suffer from comorbid conditions, including oppositional defiant disorder (ODD), conduct disorder (CD), and internalizing disorders (IDs) such as anxiety and mood disorders [5].

In general, individuals with ADHD tend to be involved in a greater proportion of risky situations and behaviors in everyday life than individuals without ADHD. Specifically, those with ADHD tend to demonstrate more dangerous driving behavior [6]–[8], increased involvement in traffic accidents [9], [10], increased criminality [11], [12], more risky sexual behavior [13], [14], and increased drug abuse [15]. In addition, in their meta-analytic review, Lee, Humphreys, Flory, Liu, & Glass [16] concluded that childhood ADHD was a risk factor for the dependence on, and abuse of, nicotine, alcohol, marihuana, and cocaine later in life. Individuals with ADHD also have an increased chance to develop problem or pathological gambling, especially individuals with ADHD-C [17], individuals with severe ADHD symptoms [18], or individuals with persistent ADHD [19].

The relationship between ADHD and risky behavior may be explained by executive dysfunctioning, in particular inhibition deficits, that for many years have been the focus of ADHD models [20], [21]. In these models, it is assumed that risky behavior in ADHD is caused by impaired impulse control due to deficiencies in inhibition of prepotent responses, interference control, and the stopping of ongoing responses after feedback on errors. More recently, some models of ADHD have also incorporated motivational deficits as the core problem in ADHD [22]–[24], which are characterized by an aberrant level of sensitivity to rewards and penalties [25]. Both behavioral studies and animal-models have suggested that children with ADHD have a greater preference for immediate over delayed rewards compared to normal controls. This increased orientation towards immediate rewards is predicted by models such as the Dual Pathway Model (DPM) [22], [26], the Dynamic Developmental Theory (DDT) [24], and the Dopamine Transfer Deficit Theory (DTD) [23]. The DPM proposes that disturbances in at least two independent neural circuits can lead to ADHD. Specifically the ventrolateral and dorsolateral cortico-striatal circuitry, which subserves executive processes, and the mesolimbic (medial-prefrontal and orbitofrontal) ventral striatal circuitry, which subserves motivational processes. Disturbances in the former circuitry give rise to cognitive and behavioral dysregulation, whereas disturbances in the latter give rise to delay aversion, resulting in relatively strong preferences for smaller immediate rewards over larger delayed rewards. The DDT and DTD are both based on the assumption that ADHD is associated with a dysfunction of the midbrain dopamine system (although the exact mechanism proposed differs between the models) and not only predict an increased preference for immediate over delayed rewards, but also predicts that children with ADHD need frequent reinforcement to learn optimally, show impaired learning in response to reinforcement, and show an impaired integration of earlier experiences of reinforcement when planning and carrying out behaviors. Several other models predict that children with ADHD also suffer from a reduced sensitivity to punishment or non-reward, which makes them more focused on rewarding stimuli than children without ADHD [27]–[29]. However, there is also evidence for reduced psychophysiological sensitivity to rewards and penalty in individuals with ADHD [30]–[32], but according to the literature review by Luman and colleagues these results are inconsistent [25]. This inconsistency in research findings is presumably caused by the many factors that influence decision-making in ADHD, such as characteristics of the individuals and the adopted task paradigm.

To gain more insight into the relationship between ADHD and risky behavior, cognitive tasks with a gambling component can be used to investigate the risky behavior of individuals with ADHD. In gambling tasks, participants can usually choose between several options that differ in the chance for a reward or penalty. The exact probability distribution of the outcome can be evident for the participant (explicit) or not (implicit). Examples of implicit gambling tasks are the Balloon Analogue Risk Task (BART) [33], the Card Playing Task (CT) [34], the Door Opening Task (DOT) [35], and the Iowa Gambling Task (IGT) [36] (see Methods section for a more detailed description of implicit gambling tasks). With regard to the IGT, which is one of the most often used paradigms, two phases of decision-making can be distinguished [37]. In the initial phase, the consequences of the decision are completely undefined and participants do not have any information about how likely positive or negative consequences will appear and, therefore, decision-making in this phase is called ambiguous. In the second phase, however, participants have some abstract knowledge of the consequences and the associated probabilities of their choices. Decisions in this phase are commonly referred to as ‘decisions under risk’. In explicit gambling tasks, the exact probability of receiving a reward or penalty is made explicit or can easily be deduced, and decisions on these types of tasks are also considered to be under risk. Examples of explicit gambling tasks are the Cambridge Gambling Task (CGT) [38], the Game of Dice Task (GDT) [39], the Make-a-Match Game (MMG) [40], and the Probabilistic Discounting Task (PD) [41] (see Methods section for a more detailed description of explicit gambling tasks).

Implicit and explicit gambling tasks aim to measure different types of decision-making. Implicit gambling tasks are thought to depend on ‘hot’ decision-making involving emotional and affective responses to the options of choice as well as on ‘cold’ decision-making involving the rational and cognitive determinations of risks and benefits associated with the options in the later stages of the task [42], [43]. Explicit gambling tasks, however, are more focused on ‘cold’ decision-making strategies because the knowledge of the probability distributions can be used to rationally determine the risks and benefits of the options right from the start of the task. According to the Dual-System Explanation risky behavior is the result of a competition between ‘hot’ and ‘cold’ decision-making processes that are subserved by, respectively, a phylogenetically older affective-motivational system (comprised of subcortical and cortical midbrain dopamine systems) and a phylogenetically younger deliberative cognitive control system (comprised of the dorsal and ventral lateral prefrontal cortex and the posterior parietal cortex) [44]–[46]. Making a distinction between implicit and explicit gambling tasks may allow for conclusions on the type of decision-making that is impaired in ADHD and the underlying systems that are affected.

Studies on the gambling task performance of individuals with ADHD show mixed results, which may be caused by the use of different task paradigms and/or by sample characteristics. Therefore, the aim of this review is to gain more insight into the relationship between ADHD and risky decision-making on gambling tasks in existing research, and to identify any alternate explanatory factors that could have influenced the outcomes presented in the literature. Based on the increased sensitivity to immediate rewards and decreased sensitivity to penalties predicted by motivational models of ADHD, it is hypothesized that individuals with ADHD will display more risky behavior in gambling tasks than individuals without ADHD. Specifically, purely motivational models [23], [24], [27]–[29] would predict that risky behavior is increased on especially implicit gambling tasks because these tasks strongly depend on both ‘hot’ and ‘cold’ decision-making, which are, respectively, underpinned by affective-motivational and cognitive control systems. Risky behavior in explicit gambling tasks, which mostly depend on ‘cold’ decision-making, would, however, be less evident due to the assumed reliance on mostly cognitive control in explicit tasks. Moreover, as reinforcement learning is an important component of implicit gambling tasks, the DDT and DTD would predict reduced performance in individuals with ADHD on specifically this type of task. Purely cognitive models [20], [21] and combined cognitive-motivational models [22], [26] on the other hand would predict increased risky behavior on both implicit and explicit gambling tasks, because both types of tasks rely on the cognitive control systems that are predicted to be impaired in ADHD by these models.

The literature was searched for studies that compared individuals with ADHD to normal controls (NCs) concerning their risky performance on a gambling task. A neuropsychological approach was taken by only including studies using standardized tasks and experimentally controlled methods. Furthermore, non-experimental studies that examined decision-making in everyday life were outside the scope of this review. The studies included were searched for the following alternate explanatory variables: the type of gambling task, comorbidity (ODD/CD and IDs), methylphenidate (MPH) use, the form of reward used, and the demographic characteristics of the participants (age, sex, and intelligence and/or education level).

## Methods

### Study Selection Procedure

**Figure 1:**
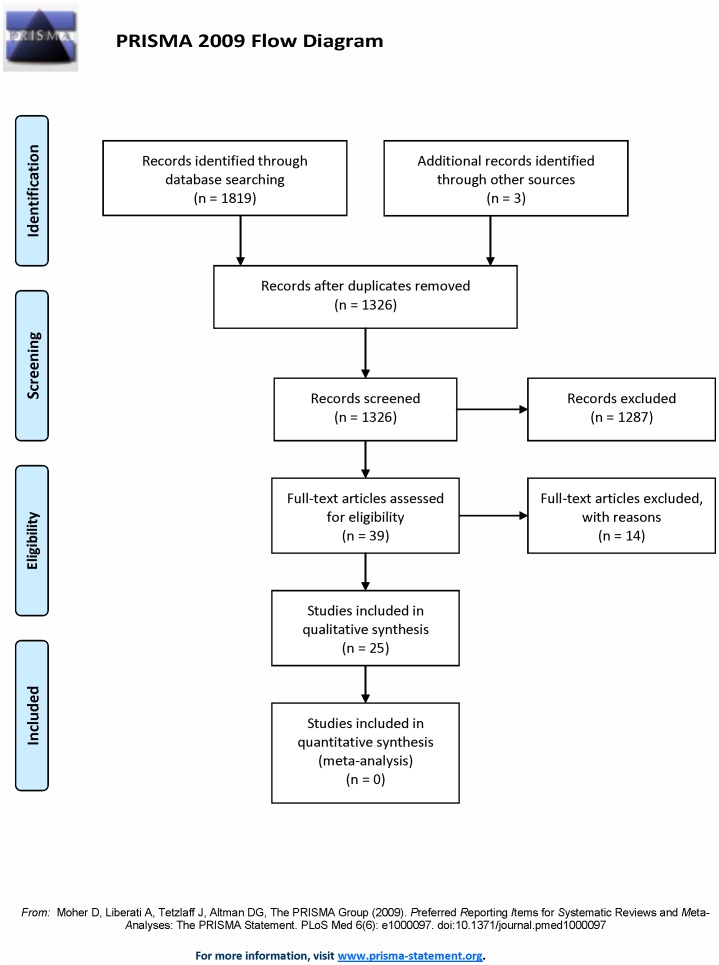
Flow diagram depicting the selection of studies according to the guidelines of Preferred Reporting Items for Systematic Reviews and Meta-Analyses (PRISMA).

This systematic literature review was carried out according to the guidelines of Preferred Reporting Items for Systematic Reviews and Meta-Analyses (PRISMA) (see Table S1 for a completed checklist of the PRISMA guidelines for this study). No protocol exists for this review. The study selection process is summarized in Figure 1. The literature was searched in PsycINFO, PubMed, and Web of Knowledge including all of the available literature up until the date of June 1, 2012. The keywords ‘ADHD’ or ‘attention deficit hyperactivity disorder’ were combined with keywords related to gambling, such as ‘risk’, ‘gambling’, ‘reward’, ‘punishment’, ‘decision-making’ and ‘probabilistic discounting’. The following selection criteria were used for the inclusion of studies: (a) the study was written in English; (b) the inclusion of both an ADHD sample and a sample of NCs; (c) a cognitive task with a gambling component was used; (d) the performance on the applied gambling task was measured in terms of risky performance. Criterion (d) means that studies that only reported reaction times or biological/physiological measures were excluded from this review. The reference lists of the initial studies were then used to trace other relevant studies. After the completion of the search 25 studies published between 1991 and 2012 were included in the review (see Table S2 for an overview of these studies).

### Identified Gambling Tasks and Outcome Measures

The studies in this review all used one or more of the following gambling tasks: the IGT or a variant of the IGT, the CT/DOT, or the BART as implicit gambling tasks, and the CGT, GDT, MMG, or PD as explicit gambling tasks. The identified gambling tasks are described below.

#### Implicit gambling tasks and outcome measures

The *Balloon Analogue Risk Task (BART)* [33] was developed to simulate risky behavior in everyday life. Risky behavior is reinforced until an implicit point in time, at which further riskiness results in poorer outcomes. In the BART, the subject is instructed to pump up a series of 90 balloons. With every pump the size (magnitude) of the balloon visibly increases and a fixed amount of money is deposited in a temporary bank, which is invisible to the subject. However, the balloons will explode after an unknown and variable number of pumps. After an explosion the money in the temporary bank will be lost and the next empty balloon will be presented. The subject can prevent an explosion by stopping the pump in time. The money in the temporary bank will then be transferred to the permanent bank, which is visible for to subject. The goal is to earn as much money as possible in the permanent bank. Examples of outcome measures are the total number of pumps, the number of pumps on the non-exploded trials (adjusted number of pumps), and the number of exploded balloons. The punishment sensitivity can be measured by subtracting the number of pumps on the trial following an exploded balloon from the number of pumps preceding an exploded balloon (post explosion reactivity).

The *Card Playing Task (CPT)* [*34*] *and Door Opening Task (DOT)* [35] were originally developed as a response perseveration task, but also contain a gambling component. In the CT, cards are sequentially presented on a screen (maximally 100 cards), with a predefined order of face cards and number cards. The face cards show a fixed reward and number cards show a fixed penalty. Unbeknownst the subject, the chance for receiving a penalty (number card) increases by 10% after each block of 10 cards, starting at 10% and then rising by 10% every 10 cards until it reaches 100%. The subject starts with a specified stake and may decide on each trial to play the card or to quit the whole game. Both quitting too soon and playing too long will result in a suboptimal outcome. The CT has several outcome measures. The total number of played cards or the number of played cards after the optimal interval (number of responses) are regarded as a measure for response perseveration, but may also be used as a measure of risky performance. The financial outcome reflects suboptimal decision-making due to early quitting or perseveration. The DOT uses the same principle as the CT. However, doors instead of cards are presented that hide a happy face (reward) or a sad face (penalty).

The *Iowa Gambling Task (IOWA)* [36] was developed to simulate real-life decision-making under uncertainty. The subjects are instructed to maximize their gain by making 100 choices (i.e. selections of cards) from four different decks of cards. They are allowed to switch decks after each selection. The subject receives a starting amount of, usually, fictive money and receives a reward for each card that is pulled, with the exception of some cards which penalize the subject. While a reward results in a gain of money, penalties take money away from the subject. On each trial, the amount of money gained or lost is presented on the screen. The four decks differ in the magnitude of the reward and in the magnitude and frequency of the penalty. Unbeknownst to the participant, the reward/penalty schedule of the cards is predefined (see Table 1). Decks A and B are regarded as the risky disadvantageous decks, because consistent card selection from these decks will lead to a net loss. Decks C and D are regarded as the safe and advantageous decks, because consistent card selection from these decks leads to a net gain. Decks A and C deliver frequent small penalties, whereas decks B and D deliver infrequent large penalties. Several outcome measures can be computed for the IGT, such as the number of choices for each separate deck, the number of safe choices, the number of risky choices, and the financial outcome. The outcome measure that is most used and reflects risky performance is the ‘net score’, which is defined as the number of selected cards from the advantageous decks minus those from the disadvantageous decks [(C+D) – (A+B)]. In order to chart the subjects’ learning effects or strategies, the outcome measures are often computed for each block of 20 trials. Several alternate variants of the IGT have also been developed.

**Table 1:** The classic reward/penalty schedule of the Iowa Gambling Task [36] for 10 successive card selections from the risky/disadvantageous decks A and B, and the safe/advantageous decks C and D.

	Card 1	Card 2	Card 3	Card 4	Card 5	Card 6	Card 7	Card 8	Card 9	Card 10
**Deck A**	+100	+100	+100	+100	+100	+100	+100	+100	+100	+100
			**−150**		**−300**		**−200**		**−250**	**−350**
**Deck B**	+100	+100	+100	+100	+100	+100	+100	+100	+100	+100
									**−1250**	
**Deck C**	+50	+50	+50	+50	+50	+50	+50	+50	+50	+50
			**−50**		**−50**		**−50**		**−50**	**−50**
**Deck D**	+50	+50	+50	+50	+50	+50	+50	+50	+50	+50
										**−250**

#### Explicit gambling tasks and outcome measures

In the *Cambridge Gambling Task (CGT)* [38] a line of ten red and blue boxes is presented on a screen, in which the number of red or blue boxes is differs each trial (with ratios of 9∶1, 8∶2, 7∶3, and 6∶4). The aim is to guess which color of box hides the reward. The subjects start with a stake of points and may, on each of 72 trials, bet on one color by selecting a proportion of their stake (which is also presented on the screen). The right color choice is rewarded with the number of points bet, whereas the wrong color choice is penalized with the same number of points bet. Several outcome measures can be computed. The quality of the performance is assessed by the proportion of trials where the majority color is chosen (rational choices). Risky behavior is represented by the overall proportion bet (amount bet) and risk adjustment is the rate at which subjects increase the bet proportion in response to more favorable ratios of red:blue boxes, with lower scores being disadvantageous.

The *Game of Dice Task (GDT)* [39] is a computerized task in which a virtual die is thrown 18 times. The aim of the task is to maximize your money by betting on the die outcome. Subjects can bet on one single die outcome with a possible reward of 1000 (1∶6 chance), or on a combination of two, three or four different die outcomes with the respective rewards of 500 (2∶6 chance), 200 (3∶6 chance) and 100 (4∶6 chance). Wrong bets lead to a penalty of the same magnitude as the possible reward (i.e. 1000, 500, 200, or 100). The options with three and four dice are regarded as the safe options, whereas the options with one or two dice are regarded as risky. Several outcome measures can be computed for the GDT, such as the number of choices for each separate option, the number of safe choices, the number of risky choices, and the financial outcome. The most often used outcome measure is the net score, which is defined as the number of safe choices minus the number of risky choices.

The Make-a-Match Game (MMG) [40] is a probabilistic discounting task that can be easily understood by children. The aim of this computerized task is to find the copy of a target card in a line of cards with their faces hidden (similar to the game memory). On each of the 12 trials, the subjects may choose from a set of two, three or four cards with the respective rewards of one (1∶2 chance), two (1∶3 chance), or three (1∶4 chance) candies when the correct card is chosen. Choosing the wrong card leads to a reward omission, but not to a direct penalty. The outcome measure is the number of choices for the three separate options or the number of candies received.

The *Probabilistic Discounting Task (PD)* [41] aims to measure the degree to which the subjective value of a large reward decreases when the probability of obtaining it decreases. Less discounting of the value of low probable (uncertain) rewards is related to risky choices. In the PD, subjects may choose on each of 120 trials between a small certain and a large uncertain reward. The magnitude of the certain reward varies from 0 to 10 cents (0, 2, 4, 6, 8, and 10), while the chance to receive it is constantly 100%. The magnitude of the uncertain reward is constant (10 cents), and varies in probability from 0 to 1 (0,.25,.50,.75, and 1). For every trial, the options are depicted by two piggy banks each containing a quantity of money. The probability of obtaining the reward is represented by the thickness of the piggy bank’s shell, and by a colored bar, in which red indicates the thickness of the shell. Pushing the button of the preferred piggy bank activates a hammer that hits it. If the piggy bank breaks, the subject receives the quantity of money in the piggy bank. The subjective values of the probabilistic rewards (which is always 10 cents) can be calculated for every probabilistic level. The subjective value of the probabilistic reward is defined as the magnitude of the small certain reward for which the participant shows indifference in a choice against the large probabilistic reward. The area under the curve (AUC) for the probabilistic discounting function can be used as the outcome measure [41]. In general, a smaller AUC reflects a steeper discounting function and more risky performance.

### Study Analysis

The results in Table S2 describe outcome measures of risky performance and the use of feedback in individuals with ADHD and NCs. A significance level of p<.05 was adopted. Effect sizes (Cohen’s d) were reported for those studies that provided the required information to compute them. However due to insufficient reporting of statistics in some of the papers, leading to missing effect sizes, and the large variation in output measures it was not possible to calculate reliable average effect sizes across studies in children/adolescents and adults. Therefore, in order to give an indication of the magnitude of the effect size the range of the effect sizes has been provided for children/adolescents and adults separately. The review was structured according to the age of individuals included in the studies (children/adolescents versus adults) and according to the type of gambling task applied (implicit versus explicit).

We identified several potential alternate explanatory variables in the literature, which were age, sex, intelligence and/or education level, ODD/CD, IDs, ADHD subtype, MPH use, and the form of reward used. The potential influences of these alternate explanatory variables are addressed in a separate section, in which a comparison was made between studies in which differences in risky behavior were found between individuals with ADHD and NCs (positive findings) and studies that did not find any group differences (null findings). A rather conservative strategy was adopted for allocating studies to the positive findings category in order to maximize generalizability. As such, studies that only found group differences for specific aspects or parts of a gambling task were allocated to the null findings category. In cases where more than one ADHD group was compared to NCs only the results of the ADHD group with the least comorbidity were used for the classification. A potential alternate explanatory variable was regarded as controlled for when the ADHD and NC samples were matched or did not differ on this variable, when statistics showed that this variable did not correlate with the performance on a particular gambling task, or when an appropriate statistical correction was carried out for the variable in question.

## Results

### Implicit Gambling Tasks in Children/Adolescents with ADHD

Ten studies investigated the performance of children/adolescents with ADHD on an implicit gambling task. Six studies used the IGT or a IGT variant [47]–[52], three studies used the DOT [35], [53], [54], and one study used the BART [55]. An overview of these studies and their results is given in Table 2.

**Table 2:** Risky performance outcomes on implicit gambling tasks in children/adolescents with ADHD.

Study: Authors (year)	Ref #	Gambling task	ADHD versus NC^a^	Risk-taking, group effects
Daugherty & Quay (1991)	[35]	DOT	+	ADHD+CD>NC
Garon et al. (2006)	[47]	Version of IGT	+	ADHD>ADHD+ID = NC
Geurts et al. (2006)	[48]	Version of IGT	−	ADHD = NC
Hobson et al. (2011)	[49]	IGT	+	ADHD>NC
Humphreys & Lee (2011)	[55]	BART	+	ADHD+ODD>ADHD>NC
Luman et al. (2008)	[50]	Variant of IGT	+/−	Magnitude condition: ADHD>NC; frequency condition: ADHD = NC
Masunami et al. (2009)	[51]	IGT	−	ADHD = NC
Matthys et al. (1998)	[53]	DOT	+	ADHD+ODD/CD>NC
Toplak et al. (2005)	[52]	IGT	−	ADHD = NC; ADHD-C = ADHD-I
Wiers et al. (1998)	[54]	DOT	−	ADHD = NC

Note: ADHD = attention deficit hyperactivity disorder; BART = Balloon Analogue Risk Task; C = combined type; CD = conduct disorder; DOT = Door Opening Task; I = inattentive type; ID = internalizing disorder (anxiety and mood disorders); IGT = Iowa Gambling Task; NC = normal control group; ODD = oppositional defiant disorder; Ref # = Reference number.

a The ADHD group with the least comorbidity was used for this comparison; (+) deviant; (+/−) partially deviant; (−) not deviant.

#### The Iowa Gambling Task (IGT)

Six studies investigated the performance of children/adolescents with ADHD on the IGT or a variant of the IGT [47]–[52], of which two studies reported that children/adolescents clearly displayed more risky behavior than NCs [47], [49]. Garon et al. [47] used a child version of the IGT and found that children with ADHD (without a comorbid ID) less often chose the advantageous decks than NCs (Cohen’s d = 1.14). The NCs also made more advantageous decisions as the task progressed, whereas the children with ADHD (without a comorbid ID) did not show this pattern, and did not choose the advantageous decks more often than predicted by chance. Hobson et al. [49] examined the performance of adolescents with ADHD on the second phase of the IGT, i.e. the risky decision-making phase in which the participants have some abstract knowledge of the riskiness of their choices (see the description in the introduction), and also found that individuals with ADHD made more risky choices than the NCs (Cohen’s d = 0.69).

Luman et al. [50] used a variant of the IGT with three options, one advantageous option (small rewards/small punishments) and two disadvantageous options (large rewards/large penalties and small rewards/large penalties). The participants performed the task in two conditions; in the ‘magnitude condition’ the magnitude of the penalty of the disadvantageous decks increased with task progression, whereas in the ‘frequency condition’ the frequency of the penalty of the disadvantageous decks increased with task progression. The results demonstrated that in the frequency condition, both the children with ADHD and the NCs showed a preference for the advantageous deck. However, in the magnitude condition, only the NCs had a preference for the advantageous deck, whereas the children with ADHD did not. The authors, therefore, presumed that children with ADHD are sensitive to the frequency, but blind to the magnitude of a punishment. Contrary to expectations, the children with ADHD did not show a particular specific preference for the disadvantageous deck with large rewards. Also, the group effect during the second task session was reduced, suggesting that children with ADHD do learn from previous experiences.

Three studies found no abnormalities in the degree of risk-taking on the IGT in children/adolescents with ADHD [48], [51], [52]. Geurts et al. [48] used a children’s variant of the IGT [56] with two conditions: the ‘standard condition’ (which is the default IGT) and the ‘reversed condition’. In the standard condition, the rewards are constant and the penalties are unpredictable, whereas in the reversed condition the penalties are constant and the rewards are unpredictable. The study revealed no differences between children with ADHD and NCs in net score (Cohen’s d = 0.04). Both groups more often chose the advantageous decks as the task progressed with this pattern emerging sooner in the reversed condition. The two groups also did not differ in the use of feedback from the previous trial, as they both changed deck more often after receiving a penalty than after a reward. Masunami et al. [51] examined decision-making patterns and sensitivity to rewards and penalties on the IGT in children with ADHD. The authors did not find abnormalities in the number of advantageous choices. However, they found differences between children with ADHD and NCs in the so-called T-patterns that are related to the sensitivity to rewards and penalties. T-patterns are pairs of events, in this case the outcomes and choices of children, which are repeated in the same order with a fixed time interval. An example of a returning T-pattern is if a child receives a penalty from deck disadvantageous deck A, then selects from safe deck C but the penalty appears in disadvantageous deck B, and the child then selects disadvantageous deck B. The results showed that there were significantly less T-patterns including penalties in children with ADHD compared to NCs, which indicates that children with ADHD paid less attention to penalties than the NCs. Toplak et al. [52] investigated the performance of adolescents with ADHD on the IGT. No group differences were found in the net score and financial outcome of the ADHD group compared to the NCs. Visual inspection demonstrated that card selections were random in the first ambiguous phase (<50 trials) in both groups. However, in the second, risky, phase adolescents with ADHD chose the disadvantageous deck with infrequent penalties more often and chose the advantageous deck with infrequent penalties less often when compared to NCs. There were no group differences in the choices for the two decks with frequent penalties, with both ADHD individuals and NC’s more often selecting the advantageous deck in this case. This supports the idea that individuals with ADHD are more sensitive for the frequency than the magnitude of penalties. Additionally, two ADHD subtypes (ADHD-C and ADHD-I) were compared. No difference was found in net score between these two subtypes of ADHD. However, the adolescents with ADHD-C chose the decks with infrequent penalties more often and the decks with frequent penalties less often compared to those with ADHD-I. Individuals with ADHD-C appear therefore to be more sensitive to the frequency and less sensitive for the magnitude of penalties in comparison to individuals with ADHD-I.

As mentioned above, Garon et al. [47] reported that children with ADHD without an ID made less advantageous choices on a child version of the IGT than NCs. This study also included a group of children with ADHD and anxiety/depression, who made significantly more advantageous choices than the ADHD group without anxiety/depression (Cohen’s d = 1.00). The children with ADHD and anxiety/depression also did not differ from the NCs (Cohen’s d<0.38) and as the task progressed they made more advantageous choices. The authors, therefore, assumed that in children with ADHD an ID has a protective effect on reinforcement learning. Another possibility they suggested is that fear, which is often increased in those with anxiety/depression, leads to an increased awareness of which decks are better or worse. Finally, as mentioned above, Hobson et al. [49] found that adolescents with ADHD displayed more risky behavior in the IGT than NCs. Additionally dimensional analyses (multiple regression analyses) revealed that ODD/CD but not ADHD symptoms were associated with risky behavior on the IGT.

#### The Door Opening Task (DOT)

Three studies investigated the performance of children/adolescents with ADHD on the DOT [35], [53], [54]. Two out of the three studies reported that children with ADHD and comorbid ODD/CD played the task longer and therefore ran more risks than the NCs [35], [53] (Cohen’s d respectively = 0.97 and 1.32). Conversely, Wiers et al. [54] found no difference in the number of played doors between children with ADHD (without comorbid ODD/CD) and NCs (Cohen’s d = 0.18).

#### The Balloon Analogue Risk Task (BART)

Humphreys & Lee [55] examined risky behavior and sensitivity to punishment on the BART in children with ADHD with and without comorbid ODD and NCs. The study showed that the ADHD group *with* comorbid ODD ran more risks by pumping up the balloons more than the ADHD group *without* comorbid ODD, who did, however, still pump the balloons more than the NCs. Contrary to expectations, the children with ADHD and comorbid ODD were most sensitive to punishment, in that they pumped the balloon less in trials after having just been penalized with a balloon pop, followed by the NCs, and then the children with ADHD without ODD, who were the least sensitive to punishment. The authors therefore assumed that children with ADHD and comorbid ODD are characterized by poor affect regulation, which makes them too reactive and/or unable to cope adequately with punishment. The authors further hypothesized that this caused children with ADHD and comorbid ODD to perform inconsistently on the gambling task, thereby demonstrating an increase in risky behavior and an increase in the frequency of impulsive adjustments of behavior after receiving penalties.

### Explicit Gambling Tasks in Children/Adolescents with ADHD

Four studies investigated the performance of children/adolescents with ADHD in explicit gambling tasks. All of which made use of a different task paradigm (CGT, GDT, MMG, and PD) [40], [41], [57], [58]. An overview of these studies and their results is given in Table 3.

**Table 3:** Risky performance outcomes on explicit gambling tasks in children/adolescents with ADHD.

Study: Authors (year)	Ref #	Gambling task	ADHD versus NC^a^	Risk-taking, group effects
DeVito et al. (2008)	[57]	CGT	−	ADHD = NC; ADHD-PL>ADHD-MPH
Drechsler et al. (2008)	[58]	GDT	+	ADHD>NC
Drechsler et al. (2010)	[40]	MMG	+	ADHD>NC
Scheres et al. (2006)	[41]	PD	−	ADHD = NC

Note: ADHD = attention deficit hyperactivity disorder; CGT = Cambridge Gambling Task; GDT = Game of Dice Task; MMG = Make-a-Match Game; MPH = methylphenidate; NC = normal control group; PD = Probabilistic Discounting Task; PL = placebo; Ref # = Reference number.

a (+) Deviant; (−) not deviant.

#### The Cambridge Gambling Task (CGT)

DeVito et al. [57] investigated the performance of children with ADHD on the CGT in a double-blind placebo-controlled within-subjects trial of MPH. In the placebo condition, the children with ADHD did not differ from the NCs on the mean betting proportion (risk-taking; Cohen’s d = 0.27). However, children with ADHD made less rational choices and scored lower on risk adjustment than the NCs. In the MPH condition, the children with ADHD bet fewer points, thereby lowering their risk, but did not differ from the placebo condition in their number of rational choices or risk adjustment.

#### The Game of Dice Task (GDT)

Drechsler et al. [58] investigated risky behavior on the GDT in children with ADHD. The children played the GDT twice. No differences between children with ADHD and NCs were found in the first game (Cohen’s d = 0.05), but children with ADHD displayed more risky behavior than NCs during the second game (Cohen’s d = 0.83). Specifically, in the second game, children with ADHD chose the most risky alternative (one die) more often than during the first trial. This poorer performance on the second trial means that if the overall performance on the first and second game is examined then the children with ADHD performed worse overall than the NCs, Based on these findings the authors suggested that children with ADHD respond to feedback in a similar fashion as NCs when confronted with something new, but show aberrant behavior when they become more used to the task.

#### The Make-a-Match Game (MMG)

Drechsler et al. [40] developed the MMG and demonstrated that children with ADHD had a greater preference for conditions with a low probability large reward, than NCs (four-card selections; Cohen’s d = 1.20). Both groups did not change their strategy during task progression and switched set equally often following positive or negative feedback. The authors explain this lack of learning effects by the absence of explicit punishments for incorrect choices in the MMG, and the fact that in this study there was no difference in the final reward that was obtained for a cautious or more risky strategy. The authors suggested that the displayed preference for larger but less probable rewards in children with ADHD points to an additional aspect of a dysfunctional reward system. The authors argue that the findings cannot be solely explained by delay aversion or oversensitivity to immediate rewards.

#### The Probabilistic Discounting Task (PD)

Scheres et al. [41] investigated whether age and ADHD symptoms affected choice preferences in children (6 to 11 years) and adolescents (12 to 17 years) on the PD. No differences between children and adolescents with ADHD and NCs were found in the area under the curve (AUC) of the probabilistic discounting function (see Methods section for an explanation of this outcome measure), indicating that both groups ran similar risks in this task (Cohen’s d = 0.27). Also, there was neither an age effect nor an interaction effect of age and diagnosis, and all groups made choices that maximized the total gain. The authors ascribed these null findings among other things to the use of explicit chances in the task design and hypothesize that individuals with ADHD have poor learning of risks, which is best measured with gambling tasks in which the chances are implicit and have to be learned.

### Summary of the Results for Children/Adolescents with ADHD

Fourteen studies investigated the performance of children/adolescents with ADHD on various gambling tasks. The effect sizes of the group differences in these studies ranged from a Cohen’s d of 0.04 to a d of 1.32. Ten studies used an *implicit gambling task*, of which five studies (5/10 = 50%) found clear evidence that children/adolescents with ADHD displayed more risky behavior than NCs [35], [47], [49], [53], [55]. An additional study only reported aberrantly risky behavior in children with ADHD in one condition (the magnitude condition) on a variant of the IGT, but not in the other condition (the frequency condition) [50]. Two of the fourteen studies investigated the effects of comorbid conditions, and found that children with ADHD and comorbid ODD/CD performed in a more risky fashion than children with ADHD without comorbidity [55]. However, children with ADHD and a comorbid ID (anxiety/depression) performed in a less risky fashion than the children with ADHD without comorbidity, who could not be differentiated from the NCs [47]. Another study compared different subtypes of ADHD and reported no differences in risky behavior between adolescents with ADHD-C and ADHD-I [52]. However, the adolescents with ADHD-C did choose decks with infrequent penalties in the IGT more often and the decks with frequent penalties less often than those with ADHD-I. Four of the fourteen studies with children/adolescents used an *explicit gambling task* and two studies (2/4 = 50%) found that children/adolescents with ADHD performed in a more risky fashion than NCs [40], [58]. Finally, another study demonstrated that MPH reduced the number of points bet in the CGT, which indicates that fewer risks were run by children/adolescents with ADHD who were treated with MPH [57]. In summary, half of the studies with children/adolescents (7/14 = 50%) found evidence for more risky behavior on gambling tasks in children/adolescents with ADHD compared to NCs, independently from the type of gambling task used (implicit or explicit).

With regard to the sensitivity to rewards and penalties (feedback use) in children/adolescents, one study found significantly less T-patterns that included penalties in the IGT in children with ADHD compared to NCs [51]. Another study reported that children with ADHD scored lower on post explosion reactivity on the BART than NCs, whereas children with ADHD with comorbid ODD scored higher on this measure than the NCs [55]. Lastly, two other studies found no differences in the number of switches after negative or positive feedback in the MMG between children with ADHD and NCs [40], [48].

### Implicit Gambling Tasks in Adults with ADHD

Eight studies investigated the performance of adults with ADHD on implicit gambling tasks. Six of these studies used the IGT or a variant of the IGT [59]–[64], two studies used the BART [64], [65], and one study used the CT [66]. An overview of these studies and their results is given in Table 4.

**Table 4:** Risky performance outcomes on implicit gambling tasks in adults with ADHD.

Study: Authors (year)	Ref #	Gambling task	ADHD versus NC^a^	Risk-taking, group effects
Agay et al. (2010)	[59]	FPGT & IGT	+/−	FPGT: ADHD>NC; IGT: ADHD = NC
Duarte et al. (2012)	[60]	IGT	−	ADHD+MA+WM>ADHD+MA = NC+/−WM
Ernst et al. (2003)	[61]	IGT	−	ADHD = NC
Fischer et al. (2005)	[66]	CT	−	Persistent ADHD = Remittent ADHD = NC; ADHD+CD>ADHD
Malloy-Diniz et al. (2007)	[62]	IGT	+	ADHD>NC
Malloy-Diniz et al. (2008)	[63]	IGT	+	ADHD>NC
Mäntylä et al. (2012)	[64]	BART & IGT	−	BART: ADHD = NC; IGT: ADHD = NC
Weafer et al. (2011)	[65]	BART	−	ADHD = NC

Note: ADHD = attention deficit hyperactivity disorder; BART = Balloon Analogue Risk Task; CD = conduct disorder; CT = Card Playing Task; FPGT = Foregone Payoff Gambling Task; IGT = Iowa Gambling Task; MA = methamphetamine dependence; NC = normal control group; Ref # = Reference number; WM = working memory impairment.

a The ADHD group with the least comorbidity was used for this comparison; (+) deviant; (+/−); partially deviant; (−) not deviant.

#### Iowa Gambling Task (IGT)

Of the six studies investigating the performance of adults with ADHD on the IGT [59]–[64], two studies reported that adults with ADHD clearly performed in a more risky manner than the NCs [62], [63]. Specifically, Malloy-Diniz et al. [62], [63] examined two different samples of adults with ADHD and found that in comparison to NCs, adults with ADHD obtained a lower net score on the standard IGT (Cohen’s d respectively = 0.79 and 0.70). The authors suggested that this was because individuals with ADHD have difficulties learning from previous experiences.

Conversely, Agay et al. [59] found no aberrant performance of adults with ADHD on the standard IGT. However, they did observe an increase in the risky behavior of their participants in the IGT variant called the ‘Foregone Payoff Gambling Task’ (FPGT). The FPGT is different from the classic form of the IGT in that not only is the outcome of the chosen card presented in the FPGT but also the outcomes of the unselected cards of the other three decks. This provides the participant with extra information, but may also distract attention of the participants. In the FPGT, adults with ADHD chose the disadvantageous decks more often than NCs. The authors suggested that the suboptimal performance of adults with ADHD on the FPGT is due to higher distractibility, and problems with divided and selective attention in the ADHD participants. The Agay et al. [59] study also examined the effects of MPH by applying a placebo-controlled ‘between-subjects’ design in which both adults with ADHD and NCs received either MPH or a placebo. No effects of MPH were found on the performance of adults with ADHD or in NCs on the standard IGT or the FPGT.

Much like Agay et al. [59], two other studies have also reported no greater levels of risky performance in adults with ADHD on the standard IGT when compared to NCs [61], [64], and one other study only revealed aberrant performance on the standard IGT in a subgroup of adults with ADHD with both hard drug dependence and working memory problems [60]. Furthermore, a study by Ernst et al. [61] also did not find any differences in the net score on the standard IGT between adults with ADHD and NCs (Cohen’s d = 0.08). However, Positron Emission Tomography (PET) analyses of the participants in the Ernst et al. [61] study did reveal the involvement of different neural networks (in particular the anterior cingulate, hippocampus, and insula) subserving emotion and memory processing in adults with ADHD as compared to the NCs during the performance of the IGT. The study of Mäntylä et al. [64] initially appears different from those described above in that they found that adults with ADHD earned less money on a standard IGT than NCs (Cohen’s d = 0.56). This group effect, however, appears to have been mediated by the educational level of the participants. Finally, Duarte et al. [60] investigated the IGT performance of adults with ADHD and a comorbid methamphetamine dependence (MA). The results indicated that only adults with ADHD+MA who also had working memory problems selected the disadvantageous decks more often than both adults with ADHD+MA *without* working memory problems and NCs both with and without working memory problems (1.94<Cohen’s d<2.04).

#### Balloon Analogue Risk Task (BART)

Two studies investigated the performance of adults with ADHD on the BART and neither study revealed significant differences in risky performance between adults with ADHD and NCs [64], [65]. Specifically, while in the study of Mäntyla et al. [64] the adults with ADHD pumped the balloons more up than NCs during the first of 10 trials, there were no group differences in the remaining 50 trials, resulting in no overall group difference on this task. Similarly, Weafer et al. [65] did not find any group differences in the total number of pumps on the BART between adults with ADHD and NCs (Cohen’s d = 0.14).

#### Card Playing Task (CT)

Only one study assessed the performance of adults with ADHD on the CT [66]. In this study, adults with persistent ADHD, adults with remittent ADHD (only ADHD in childhood), and the NCs, did not differ from each other in the number of played cards (0.01<Cohen’s d<0.24). Adults with persistent or remittent ADHD *with* a comorbid CD, however, played longer (persevered) compared to adults with persistent or remittent ADHD *without* a comorbid CD (Cohen’s d = 0.43).

### Explicit Gambling Tasks in Adults with ADHD

Three studies investigated the performance of adults with ADHD using an explicit gambling task, the GDT [67], [68] (reference 67 describes 2 separate studies). An overview of these studies and their results is given in Table 5.

**Table 5:** Risky performance outcomes on explicit gambling tasks in adults with ADHD.

Study: Authors (year)	Ref #	Gambling task	ADHD versus NC^a^	Risk-taking, group effects
Matthies et al. (2012), study 1	[67]	GDT	+	ADHD>NC
Matthies et al. (2012), study 2	[67]	GDT (boredom induction)	−	ADHD = NC
Wilbertz et al. (2012)	[68]	GDT	−	ADHD = NC

Note: ADHD = attention deficit hyperactivity disorder; GDT = Game of Dice Task; NC = normal control group; Ref # = Reference number.

a (+) Deviant; (−) not deviant.

#### Game of Dice Task (GDT)

Of the three studies assessing the GDT in adults with ADHD [67], [68], only one study found that adults with ADHD performed in a more risky fashion than the NCs [67] (study 1). Specifically, the adults with ADHD gained a lower net score than the NCs (Cohen’s d = 0.93) by tending to choose the option with two dice more often and the option with four dice less often than the NCs. The authors of this study [67] also reported differences in the way adults with ADHD and NCs made use of feedback. In that the adults with ADHD stayed less often with a safe option after positive feedback, and stayed more often with a risky option after negative feedback in comparison to the NCs. In the second study, reported in the same paper by Matthies et al. [67] (study 2), a ADHD and NC sample was assessed in which boredom was elicited by forcing the participant to wait for 5 minutes in front of a black screen before the GDT started. In contrast to the first study [67] (study 1), this study did not find any difference between adults with ADHD and the NCs in net score (Cohen’s d = 0.70) or feedback use. A direct comparison between these two studies reveals that the NCs in the boredom condition more often selected the risky options than the NCs in the condition without boredom, while the ADHD groups performed in a similar fashion in both conditions. The authors, therefore, suggested that adults with and without ADHD differ in the way they regulate boredom. This interpretation should be viewed with caution since the samples of the two studies differed (e.g. the sample of adults with ADHD in the second study scored higher on the Behavioral Inhibition Scale than the patient sample in the first study) and boredom was not assessed in the first study. In a separate study, Wilbertz and colleagues [68] did not find any differences between adults with ADHD and NCs with regard to their performance on the GDT (Cohen’s d block 1 & 2 respectively = 0.23 and 0.04). However, the ADHD and NC group did differ in their fMRI and electrodermal responsiveness to reward value. Whereas in the NCs the reward value (high versus low incentive) was differentially coded in the medial orbitofrontal cortex, this was not the case in the ADHD group. This dysfunctional coding in patients correlated with risky performance in the GDT and was paralleled by physiological arousal.

### Summary of the Results for Adults with ADHD

In total, eleven studies examined the performance of adults with ADHD on various gambling tasks. The effect sizes of the group differences between adults with ADHD and NCs in these studies ranged from Cohen’s d 0.01 to 2.04. Eight studies used *implicit gambling tasks*, of which two studies (2/8 = 25%) provided clear evidence that adults with ADHD performed in a more risky fashion than NCs [62], [63]. Another study provided mixed evidence for differences between the performance of ADHD individuals and NCs, in that only performance on the FPGT but not on the standard IGT was aberrant in adults with ADHD [59]. In addition to looking at the differences between ADHD adults and NCs, one study investigated the effects of ODD/CD comorbidity and found that adults with ADHD and comorbid CD produced more risky choices than adults with ADHD without comorbidity [66]. Only one study out of these eight that examined implicit gambling tasks investigated the effects of MPH on gambling task performance in adults with ADHD and found no evidence for an effect of MPH [59]. Finally, the performance of adults with ADHD on an *explicit gambling task* (GDT) was examined in three studies. While one study observed (1/3 = 33%) increased risky behavior in adults with ADHD [67] (study 1), the other two studies failed to find any overall group differences between adults with ADHD and NCs. Therefore, summarizing the findings of studies in adults it appears that only a minority (3/11 = 27%) of the studies found evidence that adults with ADHD compared to NCs perform in a more risky fashion on gambling tasks, a finding that appears to be independent of the type of gambling task used (implicit or explicit).

In terms of the impact of feedback, only one two-part study investigated feedback use in adults with ADHD. In their first study, Matthies et al. [67)] (study 1 found that adults with ADHD compared to NCs stayed less often with a safe option after positive feedback and stayed more often with a risky option after negative feedback. However, in their second study, in which boredom was induced before performing the gambling task, there were no differences in feedback use between adults with ADHD and NCs [67] (study 2).

### Potential Alternate Explanatory Factors

As shown above, the findings on risky decision-making in gambling tasks in individuals with ADHD are inconsistent. Several alternate factors that might influence the outcomes of the gambling tasks have already been addressed in this review that may explain this inconsistency, including type of gambling task, comorbidity (ODD/CD in children/adolescents and IDs), ADHD subtype, MPH use, the form of the reward received, and demographic factors (age, sex, and intelligence or educational level).

**Figure 2:**
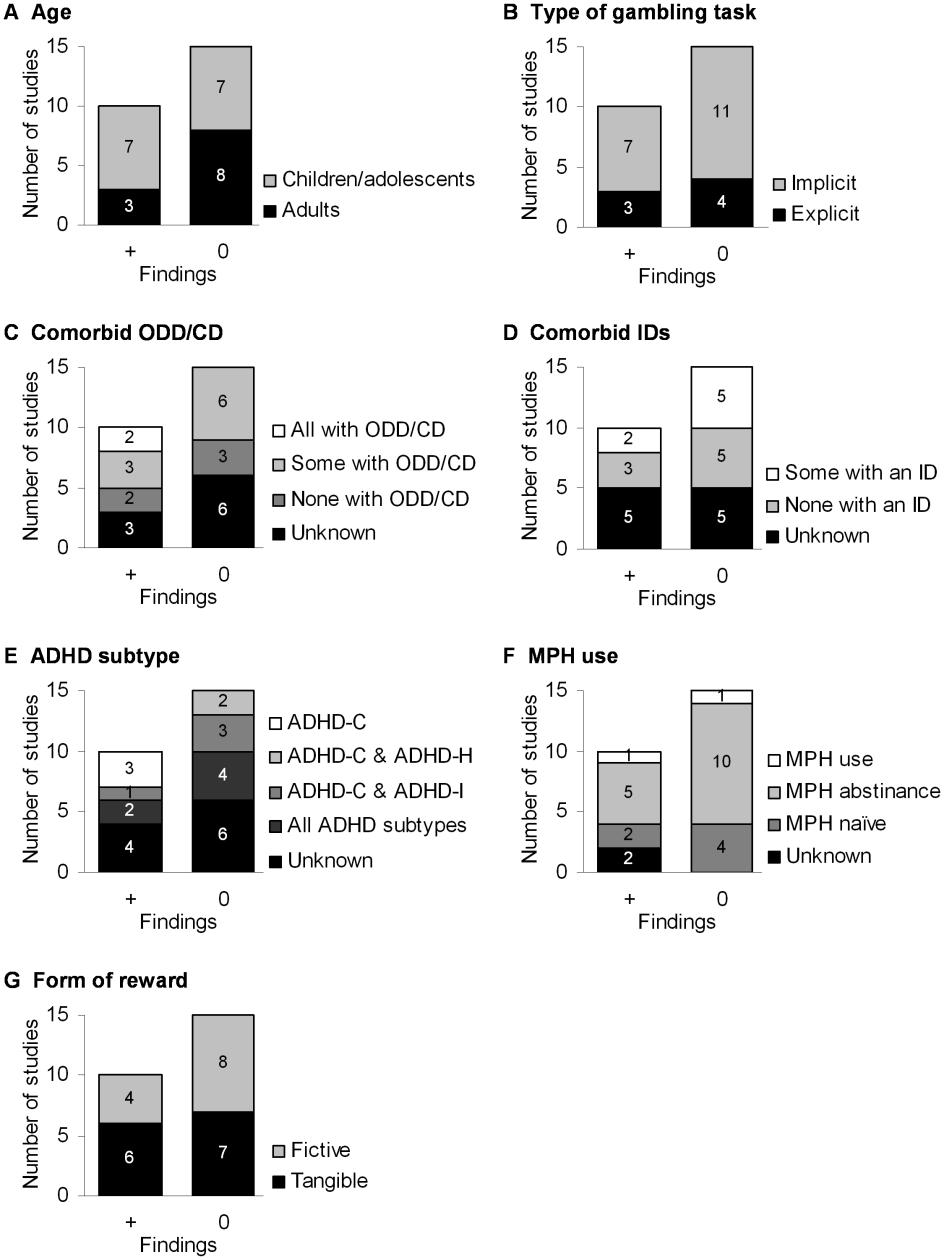
Stacked bar charts depicting the presence and absence of alternate explanatory factors split for studies with positive findings (+, i.e. increased risk-taking performance in ADHD compared to NCs) and studies with null findings (0, i.e. no ADHD-NC difference in risk-taking performance). A) Age (children/adolescents, adults); B) Type of gambling task (implicit, explicit); C) Comorbid oppositional defiant disorder/conduct disorder (ODD/CD) (all participants with comorbid ODD/CD, some participants with comorbid ODD/CD, no participants with comorbid ODD/CD, ODD/CD comorbidity unknown); D) Comorbid internalizing disorders (IDs) (some participants with a comorbid ID, no participants with a comorbid ID, ID comorbidity unknown); E) ADHD subtype (ADHD combined type only (ADHD-C), ADHD combined type and ADHD hyperactive-impulsive type (ADHD-C & ADHD-H), ADHD combined type and ADHD inattentive type (ADHD-C & ADHD-I), all ADHD subtypes, ADHD subtype unknown); F) Methylphenidate (MPH) use (MPH use during task, MPH abstinence during task, MPH-naïve participants, MPH use unknown); G) the form of reward used (fictive, tangible).

In this section, studies that provide evidence for more risky performance of individuals with ADHD in comparison to NCs, i.e. studies with positive findings, will be contrasted with regard to the aforementioned alternate explanatory variables, with studies that failed to find such evidence, i.e. studies with null findings. We made a broad categorization of studies with positive findings [35], [40], [47], [49], [53], [55], [58], [62], [63], [67] (reference 67 refers to study 1) and studies with null findings [41], [48], [50]–[52], [54], [57], [59]–[61], [64]–[66], [67], [68] (reference 67 refers to study 2). This approach was aimed at gaining insight into the factors that may cause the inconsistency of findings that appear to dominate this area. A graphical depiction of the comparison of the studies with positive and null findings for each of the alternate explanatory factors is given in Figure 2.

The first potential alternate explanatory factor is the *age of the participants*, see Figure 2A. Fourteen studies were conducted in children/adolescents. Half of these studies revealed a group effect [35], [40], [47], [49], [53], [55], [58], whereas the other half reported null findings [41], [48], [50]–[52], [54], [57]. Conversely, of the eleven studies in adults, only three studies found a group effect [62], [63], [67] (reference 67 refers to study 1) and eight studies had null findings [59]–[61], [64]–[66], [67], [68] (reference 67 refers to study 2). Therefore, the evidence for more risky performance in gambling tasks in individuals with ADHD compared to NCs appears to be stronger for children/adolescents (7/14 = 50% of studies in children/adolescents had positive findings) than for adults (3/11 = 27% of studies in adults had positive findings). In terms of the effect of age within the individuals studies, it is unlikely that age contributed meaningfully to the outcomes of the studies, because all studies except one [65] controlled for age by group matching or statistical correction (24/25 = 96% of studies controlled for age as an alternate explanatory factor).

A second potential alternate explanatory factor is the *sex* of the participants. Twenty-one out of the 25 studies examined matched their samples on sex or had equal sex ratios in the ADHD and control groups. Of the remaining studies, one study included sex as a covariate [52] and two studies mentioned that sex did not correlate with the performance on the gambling task [51], [55]. Only one study [63], which had positive findings, included samples that differed in sex ratio but did not control for this variable. Overall, sex can therefore be regarded as well-controlled for in the majority of the studies (24/25 = 96% of studies controlled for sex as an alternate explanatory factor).

A third potential alternate explanatory factor is the *intelligence/education level* of the participants. Eleven out of 25 studies reported no differences in IQ scores between the ADHD and control groups. Of the remaining studies, three studies entered IQ as a covariate in the statistical analyses [49], [52], [54], two studies reported that IQ did not correlate with performance on the gambling task [50], [66], one study did not report the influence of IQ [65], and the last eight studies did not measure IQ [35], [51], [55], [57], [59], [64], [67]. Most of the studies that did not control for IQ, however, controlled for education level [35], [57], [59], [64], [65], [67] (7/9 = 78% of the studies not controlling for IQ controlled for education level). To summarize, the majority of the studies in this review matched their participants for IQ or education level, corrected statistically the effect of IQ, or checked for the influence of group differences in IQ (23/25 = 92% of the studies performed controlled for intelligence/education as an alternate explanatory variable).

A fourth potential alternate explanatory factor is the *type of gambling task* used, see Figure 2B. At the implicit versus explicit gambling task level it seems that there is similar evidence that in the majority of studies, no matter the task type, risky performance in individuals with ADHD is not found to be higher than NCs. From the ten studies that found a group effect, three studies applied an explicit gambling task [40], [58], [67] (3/10 = 30% of the studies with positive findings; reference 67 refers to study 1) and seven studies applied an implicit gambling task [35], [47], [49], [53], [55], [62], [63] (7/10 = 70% of the studies with positive findings). This distribution was approximately the same in the fifteen studies that were categorized as having null findings, in that four studies applied an explicit gambling task [41], [57], [67], [68] (4/15 = 27% of the studies with null findings; reference 67 refers to study 2) and eleven studies applied an implicit gambling task [48], [50]–[52], [54], [59]–[61], [64]–[66] (11/15 = 73% of the studies with null findings). Inspection of the gambling tasks applied within the implicit and explicit categories revealed that the same tests were performed (IGT, CT/DOT, BART, and GDT) in studies that found a group effect as well as in studies reporting null findings. Therefore, the inconsistencies of findings cannot be attributed directly to the different types of gambling tasks or the specific tasks used for the assessment of risky decision-making. Although, possible variations between studies in how these tasks were delivered and presented cannot be ruled out.

A fifth potential alternate explanatory factor is *psychiatric comorbidity*, such as ODD/CD (see Figure 2C) and IDs (see Figure 2D). Two studies in this review compared children with ADHD and comorbid ODD/CD with individuals with ADHD without ODD/CD in their performance on the DOT, and found that the groups with comorbidity performed in a more risky fashion than the groups without comorbidity [55], [66]. Of the ten studies that demonstrated group differences in risky performance, two studies included individuals with ADHD who all had comorbid ODD/CD [35], [53] (2/10 = 20% of the studies with positive findings). Group differences between ADHD groups and NCs were also found in studies with mixed ADHD samples consisting of both individuals with and without comorbid ODD/CD [47], [49], [58] (3/10 = 30% of studies with positive findings), for studies that included individuals with ADHD without ODD/CD comorbidity [40], [55] (2/10 = 20% of studies with positive findings), and for studies in which ODD/CD comorbidity was not reported [62], [63], [67] (3/10 = 30% of studies with positive findings; reference 67 refers to study 1). A comparable proportion of studies with null findings included mixed ADHD samples [41], [48], [50], [52], [57], [66] (6/15 = 40% of studies with null findings), ADHD samples without ODD/CD comorbidity [54], [59], [61] (3/15 = 20% of studies with null findings), or did not report ODD/CD comorbidity [51], [60], [64], [65], [67], [68] (6/15 = 40% of studies with null findings; reference 67 refers to study 2). In summary, the four studies that included participants with both ADHD and ODD/CD consistently showed increased risky performance compared with NCs, whereas the studies including mixed ADHD samples and ADHD samples without ODD/CD comorbidity were not consistent in their findings.

One study in this review directly compared children with ADHD with and without a comorbid ID and found that IDs in children with ADHD lead to less risky behavior on a gambling task [47]. However, comparing studies with group effects and studies with null findings with regard to the inclusion of individuals with IDs resulted in an inconsistent pattern. Three of the eight studies (3/8 = 38%) that recruited only individuals without IDs found group differences [35], [47], [53], whereas the other five studies (5/8 = 63%) did not [50], [54], [57], [59], [61]. Similarly, only two out of the seven studies (2/7 = 29%) including a mixed sample with individuals with ADHD with and without comorbid IDs found group differences [62], [67] (reference 67 refers to study 1). The remaining five studies (5/8 = 71%) did not find any differences [41], [52], [60], [67], [68] (reference 67 refers to study 2). Ten studies did not mention the presence of comorbid IDs in their sample, of which one half had positive [40], [49], [55], [58], [63] and the other half had null findings [48], [51], [64]–[66]. In summary, one study which included children with ADHD and comorbid IDs reported less risky performance, whereas the studies that included mixed samples and ADHD samples without comorbidity were inconsistent in their results.

A sixth potential alternate explanatory factor might be the *subtype of ADHD of the participants*, see Figure 2E. Only one study in this review directly examined whether individuals with ADHD of different subtypes differed with regard to their performance on gambling tasks [52]. Comparisons within this study [52] indicated that adolescents with ADHD-C and ADHD-I were similar on the IGT in terms of their risky performance. However, the adolescents with ADHD-C more often chose the options with infrequent penalties and less often chose the options with frequent penalties when compared to the adolescents with ADHD-I. If findings are compared across studies, higher levels of risky performance were found in mixed samples of individuals with ADHD compared to NCs, which included studies that examined participants of all three ADHD subtypes [40], [58] (2/10 = 20% of studies with positive findings) or participants with ADHD-C and ADHD-I [62] (1/10 = 10% of studies with positive findings). Furthermore, three studies found that individuals with ADHD-C had performed in a more risky fashion than the NCs [35], [47], [63] (3/10 = 30% of studies with positive findings). However, there was a comparable number of studies that failed to demonstrate any difference in decision-making from NCs in mixed samples of participants with ADHD. These mixed samples included studies examining participants of all three ADHD subtypes [41], [48], [50], [60] (4/15 = 27% of studies with null findings), participants with ADHD-C and ADHD-H [52], [54] (2/15 = 13% of studies with null findings) as well as participants with ADHD-C and ADHD-I [51], [61], [68] (3/15 = 20% of studies with null findings). The findings of ten additional studies could not be taken into consideration in this discussion, since no information was provided about the participants’ subtypes of ADHD ([49], [53], [55], [57], [59], [64]–[66], [67] study 1&2). In conclusion, whereas one study reported subtle differences in risky performance between ADHD-C and ADHD-I, no consistent pattern emerged in the outcomes of studies that included samples with different subtypes and many studies did not provide enough details to be able to control for this factor.

A seventh potential alternate explanatory factor is the treatment of ADHD symptoms by using *stimulant drug treatment* (e.g. MPH), see Figure 2F. Two studies directly investigated the effects of MPH by using a placebo-controlled design. While one study found that children with ADHD on MPH performed in a less risky fashion on a gambling task compared to when on a placebo [57], the other study revealed no effects of MPH on the performance on a gambling task in adults with and without ADHD [59]. An inconsistent pattern also emerged when comparing studies with positive findings and null findings concerning the use of MPH during the assessment. Group differences between ADHD participants and NCs were present in studies in which participants discontinued medication treatment with MPH 8 to 48 hours before the experiment [40], [49], [53], [58], [67] (5/10 = 50% of studies with positive findings; reference 67 refers to study 1), in which participants were MPH-naïve [62], [63] (2/10 = 20% of studies with positive findings), and in which participants were on MPH treatment at the time of assessment [55] (1/10 = 10% of studies with positive findings). However, null findings were reported in a comparable proportion of studies (discontinuation of MPH treatment: 10/15 = 67% of studies with null findings [41], [48], [50]–[52], [57], [59], [61], [65], [67] (reference 67 refers to study 2; MPH-naïve patients: 4/15 = 27% of studies with null findings [54], [60], [66], [68]; and MPH treatment during assessment: 1/15 = 7% of studies with null findings [64]). The two remaining studies with positive findings unfortunately did not specify whether participants were on medication at the time of assessment [35], [47]. To summarize, no consistent effects of MPH on participant’s performances in gambling tasks were observed in the reviewed studies.

An eighth potential alternate explanatory factor is the *form of reward received by the participants*, see Figure 2G. Thirteen studies in this review explicitly mentioned that the subjects could win tangible rewards (such as presents and real money), of which six studies found a group effect [35], [40], [47], [49], [53], [55] and seven studies had null findings [41], [50], [52], [59], [61], [65], [68] (6/13 = 46% of the studies using tangible rewards had positive findings). The other twelve studies used fictive rewards (such as points or fictive money), or did not explicitly mentioned that they used real rewards [48], [51], [54], [57], [58], [60], [62]–[64], [66], [67], The results of these, fictive reward, studies were also inconsistent (4/12 = 33% of the studies using fictive rewards had positive findings).

## Discussion

The aim of this review was to gain more insight into the relationship between ADHD and risky performance in gambling tasks and to identify potential alternate explanatory factors that may have also affected the outcome. In total, 25 studies were reviewed that examined the performance of children/adolescents (14 studies) and adults (11 studies) with ADHD on a gambling task. Ten of the 25 included studies, i.e. 40%, reported that individuals with ADHD displayed more risky behavior in gambling tasks, as indicated by significantly higher scores than NCs on outcome measures related to risk-taking. In terms of potential alternate explanatory factors, age appeared to play an important role in the relationship between ADHD and risky decision-making, as half of the studies in children/adolescents (50%), but only a minority of studies in adults (27%) reported greater risky performance in individuals with ADHD when compared to NCs. Across the studies with children/adolescents and adults effect sizes ranged from small to large, with no clear pattern related to age. The results of the studies examined did not differ between studies applying explicit gambling tasks, in which the exact probability distribution is evident for the participants (such as the CGT, GDT, MMG and PDT), and implicit gambling tasks in which the exact probability distribution is not evident (such as the BART, CT, DOT, or the IGT). This review therefore provides evidence that children/adolescents with ADHD appear to be likely to perform in a risky fashion in gambling tasks than NCs (although, only 50% of the studies found this result), whereas adults with ADHD are less likely to perform differently from NCs on gambling tasks. This finding holds for both age groups irrespective of the use of an implicit or explicit gambling task.

However, as the results varied between studies it was investigated whether other alternate explanatory factors could also help explain these inconsistencies. Two studies showed consistently that the presence of comorbid ODD or CD increases the risky performance on implicit gambling tasks in children and adults with ADHD. Furthermore, two additional studies including only children with ADHD and a comorbid ODD/CD had positive findings. Children and adults with ADHD and comorbid ODD/CD could, therefore, be more prone to risky performance in implicit gambling tasks. Several other alternate explanatory factors have been reported in the literature, such as comorbid IDs, ADHD subtype, MPH use, and the form of reward used. The evidence for a substantial influence of these variables was limited and/or inconsistent. Future studies on risky behavior should therefore take these variables into account in the study design. The outcomes of this review are, however, not likely to be distorted by demographical differences between individuals with ADHD and NCs within the studies concerning age, sex, and intelligence/education, because a vast majority of studies controlled for these factors by means of group matching or statistical correction. It is unclear how limited power of the included studies may have contributed to the prevalence of null findings. The variability in effect sizes reported limits the possibility to compute the required sample size for reaching adequate power. Nevertheless, 17 of the 25 studies (68%) included ADHD samples that were smaller than n = 30, which are in general small sample sizes. Future studies should therefore include larger sample sizes to assure adequate power.

The result that children/adolescents with ADHD may be more likely to perform in a more risky fashion on gambling tasks than NCs, irrespective of the use of explicit or implicit tasks, implies both altered ‘cold’ and ‘hot’ decision-making strategies. On the one hand, more risky behavior on explicit gambling tasks implies an impaired ‘cold’ decision-making strategy, which may be due to deficiencies in the cognitive control system that is comprised of the dorsal and ventral lateral prefrontal cortex and posterior parietal cortex [44]–[46]. Important capabilities in this strategy are the understanding of probabilities, the ability to update this knowledge in working memory and store it in long-term memory, and to be able to inhibit responses to occasional feedback [69]. On the other hand, more risky behavior on implicit gambling tasks implies impaired ‘hot’ and ‘cold’ decision-making strategies, which may be due to deficiencies in the cognitive control system as well as the affective-motivational system that is comprised of the subcortical and cortical midbrain dopamine systems. Functions that are important for ‘hot’ decision-making are the processing of reward and punishment (which is also linked to inhibitory control) and the visceral responses to these motivational cues [42], [43]. There was no evidence that decision-making in ADHD was especially impaired on implicit gambling tasks, as would be predicted by purely motivational models like the DDT [24] and DTD [23]. The studies with positive results reported in this review are, however, more in line with ADHD models that predict cognitive deficits [20], [21] and with ADHD models that predict combined cognitive-motivational deficits, i.e. the DPM [22], [26]. Purely motivational models also do not explain the impaired performance on the studies which reported positive findings for explicit gambling tasks, tapping primarily ‘cold’ decision-making strategies. Although, it should be noted that the majority of studies overall did not report any impairment in ADHD participants, which is a challenge to both the cognitive and motivational models.

With regard to motivational deficiencies, the motivational models and combined motivational and cognitive models have primarily focused on the stronger discounting of future over immediate rewards (delay aversion) in individuals with ADHD. The increased likelihood of children with ADHD to perform more risky on implicit gambling tasks found in the literature, however, could point to an additional aspect of a dysfunctional reward (or punishment) system, which is that children/adolescents with ADHD favor less probable large rewards over more probable smaller rewards, and risk higher penalties for those rewards. This is in somewhat in line with the prediction of motivational models that participants with ADHD would perform poorer under partial or discontinuous reinforcement schedules, because reinforcement during gambling tasks is by definition discontinuous, i.e. a behavioral response may lead to different outcomes (although some outcomes are more probable than others). An important ability in the face of discontinuous reinforcement is the use of outcome feedback in order to subsequently adapt behavior or to change strategy. Only a few studies investigated the use of feedback and the findings were mixed. Some studies demonstrated aberrant feedback use in children/adolescents with ADHD, such as a reduced use of negative feedback [51] and a reduced adjustment of strategy after a punishment [55], [67] (reference 67 refers to study 2). Other studies failed to find any deviations in feedback use in individuals with ADHD [40], [48], [67]. Interestingly, two studies provided evidence that children/adolescents with ADHD did not differ from NCs in the number of risky decisions in conditions with relatively *frequent* punishment [50], [52]. This finding suggests that while in cases where feedback is frequent individuals with ADHD react in a similar way to their NC peers, but in cases where feedback is infrequent there are likely to be more problems. The outcomes of this literature review therefore provide some evidence that children/adolescents with ADHD do not only more strongly prefer immediate over future rewards than NCs, but also have a greater preference for less probable large rewards over more probable smaller rewards, and risk higher penalties for these larger rewards. However, there is some evidence that with more frequent penalties children/adolescents are better able to develop an advantageous strategy.

The evidence for aberrant risk-taking performance on gambling tasks is stronger for children/adolescents with ADHD than for adults with ADHD, although is it still only present in around fifty percent of the reviewed studies in children/adolescents. A possible explanation for the higher proportion of positive studies in children/adolescents is the developmental trajectory of ADHD, which is characterized by a reduction of symptoms from childhood to adulthood (often accompanied with remission of ADHD). The prefrontal recovery hypothesis [73] postulates that the reduction of ADHD symptoms during adolescence is related to the degree in which prefrontal cognitive control functions (‘cold’ decision-making) compensate for primary and persistent subcortical deficits (‘hot’ decision-making). The weaker evidence for increased risky performance in gambling tasks for adults compared to children with ADHD may therefore be due to developmental improvements in cognitive control functions. Another explanation for the different outcomes in children/adolescents and adults may, however, be that the study results in this review have been influenced by publication bias. Risky behavior on gambling tasks in children/adolescents with ADHD has been studied for roughly twenty years at the time of this review, and in those years findings indicating group differences may have been given preference for publication. Therefore, attempts to replicate the differences observed between children/adolescents with ADHD and NCs in the adult population, may have resulted in more publications reporting null findings within the past ten years. There is, therefore, a need for longitudinal or cross-sectional studies in order to directly test the hypothesis that the development from childhood to adulthood, and the related persistence or remittance of ADHD symptoms, influences the performance in gambling tasks in ADHD.

Even though MPH is the most prescribed pharmacological treatment for ADHD, only two studies investigated the effects of MPH on gambling task performance. One placebo-controlled study found that children with ADHD taking MPH bet fewer points in the CGT, indicating more conservative play. The other placebo-controlled study was carried out on adults and found no effects of MPH on performance on the IGT and FPGT in adults with ADHD or in NCs. Given the small number of studies and the inconsistent findings, no conclusions can be drawn about the effectiveness of MPH in reducing risky behavior in gambling tasks. Literature on the effects of MPH on risky behavior in the real-world, however, suggests that MPH has a beneficial effect. MPH has for example been demonstrated to reduce the risk for drug abuse [74] and risky driving behavior [75] in individuals with ADHD. The mechanisms underlying these effects are unclear, so further controlled studies on this subject are need to gain more insight into these mechanisms.

Although there might be some association between risky performance on gambling tasks and childhood ADHD, little is known about the relationship between the performance on such tasks and behavior in real life. Some of the reviewed studies in children with ADHD suggest that there is an association between risky performance on gambling tasks and the severity of ADHD symptoms [49], [52], [58]. However, such associations have also been found for ODD/CD symptoms in these children [48], [49] which is in line with the conclusion of this review that the presence of comorbid ODD/CD in ADHD increases risky behavior in gambling tasks. To the best of our knowledge no studies are available on the ecological validity of gambling task performance in children/adolescents. However, studies on adults have revealed a link between risky performance in gambling tasks and clinically relevant risky behaviors, e.g. between the performance in the IGT and substance use disorders, pathological gambling and psychopathic behavior [70], between performance in the PD and pathological gambling as well as alcohol dependence [71], [72], and between performance on the BART and self-reported occurrence of addictive, health, and safety risk behaviors [33]. These studies on adults clearly suggest that there is a relationship between risky performance in gambling tasks and real-life risky behavior, but more research is needed to firmly establish this, especially in children/adolescents.

### Conclusion

This systematic literature review on performance in gambling tasks of individuals with ADHD found mixed evidence for increased risky behavior. Specifically, in children with ADHD half of the studies showed increased risky performance when compared to NCs. In adults, the evidence was weaker, with only a minority of studies (27% of the studies in adults) finding any increase in risky behavior on gambling tasks in adults with ADHD. The effect sizes in these studies ranged from small to large for both age groups. Given this variability in effect sizes and the generally small sample sizes (n<30 in 68% of the included studies), it is unclear whether limited power has contributed to the mixed findings. It is possible that the age related difference is due to developmental changes occurring during the transition from childhood to adulthood. However, this age related pattern might also reflect a publication bias for positive findings in children/adolescents in the past twenty years of research.

Concerning the gambling tasks themselves, the outcome did not differ between studies applying implicit or explicit gambling tasks, which implies that, in the cases where risky performance was observed, both ‘cold’ and ‘hot’ decision-making strategies may have been altered in children/adolescents with ADHD. This finding cannot solely be explained by motivational models, because also ‘cold’ decision-making appears to be deficient, but is in line with the predictions of cognitive and combined motivational and cognitive models of ADHD, such as the behavioral inhibition model [20], executive functioning model [21], and the Dual Pathway Model [22], [26]. Although, these models would still struggle to explain why there are many null findings in the literature. However, given the age-related pattern, future studies should aim to elucidate the tenability of these models for adults with ADHD.

With regard to potential alternate explanatory factors, the literature indicates that the presence of ODD/CD is a risk factor in ADHD that can result in increases in risky behavior in gambling tasks. Several other potential alternate explanatory factors have been reported in the literature, including comorbid IDs, ADHD subtype, use of MPH, and the form of reward used. The evidence for a substantial contribution of these variables to the relationship between ADHD and risky decision-making was limited and/or inconsistent, especially given the prevalence of null findings in the literature. However, the outcomes of this review are not likely distorted by age, sex or intelligence/educational differences between participants with ADHD and NCs, because the majority of studies controlled for these variables.

The increased risky performance in some children/adolescents with ADHD in implicit gambling tasks provides some evidence that children/adolescents with ADHD do not only prefer immediate over future rewards, but also prefer less probable large rewards over more probable smaller rewards, and risk higher penalties for these larger rewards. However, there is also some evidence that with more frequent punishment that both children/adolescents with ADHD are better able to develop an advantageous strategy. It remains unclear, however, how increased risky behavior in gambling tasks relates to real-life decision-making, firstly because of the mixed findings in the area, but also because evidence for the ecological validity of the available gambling tasks is limited, especially in children/adolescents.

## Supporting Information

Table S1 Checklist of the guidelines of Preferred Reporting Items for Systematic Reviews and Meta-Analyses (PRISMA).
(DOC)

Table S2 Descriptives and results of studies considered in the review.
(DOC)
