# Supplementary material for: Risky Behavior in Gambling Tasks in Individuals with ADHD – A Systematic Literature Review
Source: PLoS One. 2013 Sep 13;8(9):e74909. doi: 10.1371/journal.pone.0074909 (PMC3772864; doi:10.1371/journal.pone.0074909)
Supplement: Table S2 — Descriptives and results of studies considered in the review. (DOC) [file pone.0074909.s002.doc]

**Table S2** Descriptives and results of studies considered in the review

| **Study: Authors (year) [Ref #]** | **Subjects: n group (subtypes)** | **% of boys** | **Age in years: Mean (range)** | **Controlled variables^a^** | **Gambling task** | **Differential group effects (Cohen’s d effect size)** |
| --- | --- | --- | --- | --- | --- | --- |
| **Children/adolescents** | | | | | | |
| Daugherty & Quay (1991) [35] | 10 ADHD+CD  (10-C)  15 NC | 48% | 11.2 (8-13) | Achievement, age, sex, ID | DOT (maximum of 100 trials)   - Earnings (chips) exchanged for presents (tangible reward) | Number of responses:  ADHD+CD > NC (d = 0.97)  Number of earned tokens:  ADHD+CD < NC (d = 1.40) |
| DeVito et al. (2008) [57] | 21 ADHD  (subtypes unknown)  22 NC | 100% | 10.2 (7-13) | Age, education, sex, ID | CGT (2 sessions of 72 trials each)   - The ADHD subjects performed the CGT once with PL and once with MPH - Fictive reward (points) | Amount bet:  ADHD-PL = NC (d = 0.27)  ADHD-PL > ADHD-MPH (d = 0.68)  Rational choices:  ADHD-PL < NC (d = 0.98)  ADHD-PL = ADHD-MPH (d = 0.03)  Risk adjustment:  ADHD-PL < NC (d = 0.93)  ADHD-PL = ADHD-MPH (d = 0.10) |
| Drechsler et al. (2008) [58] | 23 ADHD  (16-C, 2-H, 5-I)  24 NC | 94% | 12.0 (11-13) | Age, IQ, sex | GDT (2 games of 18 and 12 trials)   - Fictive reward (money) | Number of risky choices: ADHD > NC  (Game 1: ADHD = NC (d = 0.05); Game 2: ADHD > NC (d = 0.83))  Monetary outcome: ADHD < NC  (Game 1: ADHD = NC (d = 0.03); Game 2: ADHD < NC (d = 1.13)) |
| Drechsler et al. (2010) [40] | 28 ADHD  (20-C, 3-H, 5-I)  28 NC | 71% | 9.2 (7-10) | Age, IQ, sex, ODD/CD | MMG (2 games of 12 trials each)   - Earnings (candies) exchanged for candies (tangible reward) | Two-card selections: ADHD < NC (d = 0.83)  (Game 1: ADHD < NC; Game 2: ADHD = NC)  Three-card selections: ADHD = NC (d = 0.39)  (Game 1: ADHD = NC; Game 2: ADHD < NC)  Four-card selections: ADHD > NC (d = 1.20)  (Game 1: ADHD > NC; Game 2: ADHD > NC)  Number of earned tokens:  Game 1: ADHD = NC (d = 0.40)  Game 2: ADHD = NC (d = 0.39)  Number of shifts after feedback:  Positive feedback: ADHD = NC (d = 0.21)  Negative feedback: ADHD = NC (d = 0.07) |
| Garon et al. (2006) [47] | 10 ADHD+ID  (10-C)  11 ADHD  (11-C)  21 NC | 81% | 9.8 (6-13) | Age, IQ, SES, sex, ID, ODD/CD (as CV) | Child version of the IGT (80 trials)   - Earnings (tokens) exchanged for presents (tangible reward) | Number of safe choices:  ADHD < ADHD+ID = NC  (ADHD < NC: d = 1.14; ADHD < ADHD+ID: d = 1.00; ADHD+ID = NC: d < 0.38) |
| Geurts et al. (2006) [48] | 20 ADHD  (15-C, 1-H, 4-I)  22 NC | 83% | 10.0 (8-12) | Age, IQ, sex | Child version of the IGT  (2 versions of 200 trials each)   - Standard & reversed version - Fictive reward (apples) | Net score:  ADHD = NC (d = 0.04)  Number of switches:  ADHD = NC (d = 0.60) |
| Hobson et al. (2011) [49] | 31 ADHD  (subtypes unknown)  34 NC | 78% | 13.2 (10-17) | Age, ethnicity, IQ, sex | IGT (100 trials)   - Earnings cashed for money (tangible reward) | Number of risky choices (2^nd^ half):  ADHD > NC (d = 0.69) |
| Humphreys & Lee (2011) [55] | 48 ADHD+ODD  (subtypes unknown)  55 ADHD  (subtypes unknown)  87 NC | ≈ 71% | ≈ 7.4 (5-10) | Age, sex, ODD/CD | BART (2 versions of 30 trials each)   - Version 1: Mouse-click for each pump; Version 2: Type number of pumps - Earnings (points) exchanged for stickers (tangible reward) | Number of total pumps:  ADHD+ODD > ADHD > NC  Post explosion reactivity:  ADHD+ODD > NC > ADHD |
| Luman et al. (2008) [50] | 23 ADHD  (15-C, 3-H, 5-I)  20 NC | 77% | 9.4 (7-12) | Age, IQ, sex, ID | Variant of IGT (2 versions of 180 trials each)   - Magnitude and frequency condition - Earnings (money) exchanged for a present (tangible reward) | Number of risky choices:  Magnitude condition: ADHD > NC  Frequency condition: ADHD = NC |
| Masunami et al. (2009) [51] | 14 ADHD  (13-C, 1-I)  11 NC | 76% | 11.6 (7-14) | Age, sex | IGT (100 trials)   - Fictive reward (points) | Number of safe choices:  ADHD = NC  T-patterns:  With punishments: ADHD < NC  With rewards: ADHD = NC  Without rewards and punishments: ADHD = NC |
| Matthys et al. (1998) [53] | 10 ADHD+ODD/CD  (subtypes unknown)  31 NC | 100% | M = 9.7  SD = 1.1 | Age, IQ, sex, ID | DOT (maximum of 110 trials)   - Money cashed during task (tangible reward) | Number of responses:  ADHD+ODD/CD > NC (d = 1.32) |
| Scheres et al. (2006) [41] | 22 ADHD  (13-C, 2-H, 7-I)  24 NC | 74% | 11.4 (6-17) | Achievement, age, IQ, sex | PD (120 trials)   - Earnings (money) from practice trials cashed for money (tangible reward) | Area under the curve (AUC):  ADHD = NC (d = 0.27) |
| Toplak et al. (2005) [52] | 44 ADHD  (27-C, 17-I)  34 NC | 67% | 15.5 (13-18) | Age, IQ, sex, working memory performance | IGT (100 trials)   - Earnings (money) exchanged for gift certificate (tangible reward) | Net score:  ADHD = NC  ADHD-C = ADHD-I  Number of choices:  Risky frequent deck: ADHD = NC (d = 0.13)  Risky infrequent deck: ADHD > NC (d = 0.46)  Safe frequent deck: ADHD = NC (d = 0.05)  Safe infrequent deck: ADHD < NC (d = 0.53)  Frequent decks: ADHD-C < ADHD-I (d = 0.65)  Monetary outcome:  ADHD = NC (d = 0.38) |
| Wiers et al. (1998) [54] | 28 ADHD  (27-C, 1-H)  34 NC | 100% | 9.1 (7-11) | Age, IQ, SES, sex, ID, ODD/CD | DOT (maximum of 100 trials)   - Fictive reward (money) | Number of responses:  ADHD = NC (d = 0.18) |
| **Adults** | | | | | | |
| Agay et al. (2010) [59] | 32 ADHD  (subtypes unknown)  (16 PL & 16 MPH)  26 NC  (13 PL & 13 MPH) | 50% | 32.6 (21-50) | Age, education, sex, ID, ODD/CD | IGT and FPGT (100 trials each)   - FPGT: the subjects also get to see the outcomes of the other three cards - Earnings (money) cashed for money (tangible reward) | Net score IGT:  ADHD-PL = ADHD-MPH = NC-PL = NC-MPH  Number of risky choices FPGT:  ADHD > NC |
| Duarte et al. (2012) [60] | 23 ADHD+MA  (14-C, 7-H, 2-I)  22 NC | 91% | M = 40.5  SD = 10.7 | Age, education, ethnicity, sex, WM | IGT (100 trials)   - Fictive reward (money) | Net score:  ADHD+MA+WM < ADHD+MA = NC+/-WM  (ADHD+MA+WM < other groups: 1.94 < d < 2.04)  (No group effects for block 1, 2 & 5) |
| Ernst et al. (2003) [61] | 10 ADHD (5/5)  (6-C, 4-I)  12 NC (6/6) | 50% | 29.3 (21-45) | Age, IQ, SES, sex, ID, ODD/CD | IGT (100 trials)   - Earnings (money) cashed for money (tangible reward) | Net score:  ADHD = NC (d = 0.08) |
| Fischer et al. (2005) [66] | 68 Persistent ADHD  (subtypes unknown)  53 Remittent ADHD  (subtypes unknown)  70 NC | 90% | 20.9 (19-25) | Age, IQ, sex | CT (maximum of 100 trials)   - Fictive reward (money) | Number of responses:  Persistent ADHD = Remittent ADHD = NC  (0.01 < d < 0.24)  ADHD+CD > ADHD (d = 0.43)  Monetary outcome:  Persistent ADHD = Remittent ADHD = NC  (0.06 < d < 0.45  ADHD+CD = ADHD |
| Malloy-Diniz et al. (2007) [62] | 50 ADHD  (36-C, 14-I)  51 NC | 58% | 32.9 (18-60) | Age, education, IQ, sex | IGT (100 trials)   - Fictive reward (money) | Net score:  ADHD < NC (d = 0.79)  (No group effects for block 1 & 2) |
| Malloy-Diniz et al. (2008) [63] | 25 ADHD  (25-C)  50 NC | 44% | M = 30.8  SD = 8.0 | Age, education, IQ | IGT (100 trials)   - Half of the NCs performed the English (Eng) IGT, whereas the other subjects performed the Brazilian Portuguese (Br) IGT - Fictive reward (money) | Net score:  ADHD < NC-Br = NC-Eng  (ADHD < NC-Br: d = 0.70; ADHD < NC-Eng: d = 0.69; NC-Eng = NC-Br: d = 0.01)  (No group effects for block 1 & 2) |
| Mäntylä et al. (2012) [64] | 31 ADHD  (subtypes unknown)  32 NC | 54% | 30.3 (18-65) | Age, education, sex, depression (as CV) | IGT (100 trials)  BART (2 blocks of 30 trials each)   - Fictive reward (money) | Monetary outcome IGT:  ADHD = NC (with CVs; d = 0.56 without CVs)  Adjusted number of pumps BART:  Block 1: ADHD = NC (d = 0.14 without CVs)  (set 1: ADHD > NC; set 2 & 3: ADHD = NC)  Block 2: ADHD = NC (d = 0.01 without CVs)  (set 1-3: ADHD = NC) |
| Matthies et al. (2012), study 1 [67] | 15 ADHD  (subtypes unknown)  16 NC | 52% | M = 35.1  SD = 12.6 | Age, education, sex, depression (as CV) | GDT (18 trials)   - Fictive reward (money) | Net score:  ADHD < NC (d = 0.93 with depression as CV)  Number of choices:  One die: ADHD = NC (d = 0.65)  Two dice: ADHD > NC (d = 0.72)  Three dice: ADHD = NC (d = 0.43)  Four dice: ADHD < NC (d = 1.14)  Monetary outcome:  ADHD < NC (d = 0.86)  Feedback utilization:  Stay with safe decision after positive feedback on a safe decision: ADHD < NC (d = 0.94)  Stay with risky decision after negative feedback on a risky decision: ADHD > NC (d = 0.79)  Switch to safe decision after negative feedback on a risky decision: ADHD > NC (d = 0.85)  Feedback otherwise: ADHD = NC |
| Matthies et al. (2012), study 2 [67] | 14 ADHD  (subtypes unknown)  13 NC | 52% | M = 35.3  SD = 10.9 | Age, education, sex, depression (as CV) | GDT (18 trials)   - Boredom induction before performing the task - Fictive reward (money) | Net score:  ADHD = NC (d = 0.70 with depression as CV)  Number of choices:  One, two, three & four dice: ADHD = NC  (d = 0.08; d = 0.45; d = 0.57; d = 0.71)  Monetary outcome:  ADHD = NC (d = 0.17)  Feedback utilization:  ADHD = NC |
| Weafer et al. (2011) [65] | 30 ADHD  (subtypes unknown)  21 NC | 52% | 21.6 (19-25) | Education, sex | BART (20 trials)   - Earnings (money) cashed for money (tangible reward) | Number of total pumps:  ADHD = NC (d = 0.14) |
| Wilbertz et al. (2012) [68] | 28 ADHD  (20-C, 8-I)  28 NC | 52% | M = 36.9  SD = 9.2 | Age, education, IQ, sex | GDT (2 blocks of 12 trials each)   - Earnings (points) cashed for money (tangible reward) | Number of risky choices:  Block 1: ADHD = NC (d = 0.23)  Block 2: ADHD = NC (d = 0.04)  Negative feedback use:  Block 1: ADHD = NC (d = 0.23)  Block 2: ADHD = NC (d = 0.09) |

ADHD = attention deficit hyperactivity disorder; BART = Balloon Analogue Risk Task; C = combined type; CD = conduct disorder; CGT = Cambridge Gambling Task; CT = Card Playing Task; CV = covariate; DOT = Door Opening Task; FPGT = Foregone Payoff Gambling Task; GDT = Game of Dice Task; H = hyperactive-impulsive type; I = inattentive type; ID = internalizing disorder (anxiety and mood disorders); IGT = Iowa Gambling Task; MA = methamphetamine dependence; MMG = Make-a-Match Game; MPH = methylphenidate; NC = normal control group; ODD = oppositional defiant disorder; PL = placebo; Ref # = Reference number; SES = socioeconomic status; WM = working memory impairment.

^a^ A variable was regarded as controlled for either when the ADHD and NC samples were matched or did not differ on this variable, when statistics showed that this variable did not correlate with the performance on a particular gambling task, or when a statistical correction was carried out with this variable.
